# The association of antihistamines and new-onset osteoarthritis in patients with autoimmune disease: a population-based cohort study

**DOI:** 10.3389/fimmu.2025.1712222

**Published:** 2026-01-15

**Authors:** Chih-Feng Chang, Tzu-Hsien Chao, Jing-Yang Huang, Pei-Lun Liao, Chih-Jung Yeh, Tsung-Yuan Yang, Gwo-Ping Jong

**Affiliations:** 1 Division of Cardiology, Department of Internal Medicine, Taichung Armed Force General Hospital, Taichung, Taiwan; 2 School of Public Health, Chung Shan Medical University, Taichung, Taiwan; 3 Department of Surgery, Taichung Armed Force General Hospital, Taichung, Taiwan; 4 Division of Cardiovascular Surgery, Department of Surgery, Tri-Service General Hospital, National Defense Medical University, Taipei, Taiwan; 5 Department of Medical Research, Chung Shan Medical University Hospital, Taichung, Taiwan; 6 Department of Internal Medicine, Chung Shan Medical University Hospital, Taichung, Taiwan; 7 Institute of Medicine, Chung Shan Medical University, Taichung, Taiwan

**Keywords:** antihistamine, autoimmune disease, male, osteoarthritis, young age

## Abstract

**Background:**

Antihistamines have been reported to be linked with new-onset osteoarthritis (NOO). However, the effect of these drugs on NOO is unclear in patients with autoimmune disease. In this study, we aimed to investigate the effects of an antihistamine on NOO in patients with autoimmune disease.

**Methods:**

We conducted a retrospective population-based cohort study involving patients who received antihistamines therapy and patients who did not receive antihistamines therapy. The outcome was the risk of NOO. We used multivariable Cox proportional hazard models to calculate the hazard ratios (HRs) and 95% confidence intervals (CIs) for the associations between antihistamines use and NOO.

**Results:**

Overall, 49,078 patients who received antihistamines therapy and 196,312 patients who did not receive antihistamines therapy were matched for age, sex, and index date at a ratio of 1:4 between 2012 and 2022. During a follow-up of 9 years, 12,761 and 47,982 NOO events occurred in antihistamine users and nonusers, respectively. Antihistamine use was associated with a significantly higher risk of NOO (adjusted HR 1.05, 95% CI: 1.03–1.07, *P* < 0.0001). We confirmed the robustness of these results through a propensity score 1:1 matching analysis.

**Conclusions:**

In this population-based cohort study, we found that antihistamine use was associated with a significantly increased risk of osteoarthritis in patients with autoimmune disease. Further efforts are needed to minimize the potential population drawbacks of these therapies in high-risk groups.

## Introduction

Osteoarthritis and autoimmune disease are well established as prevalent diseases with significant associated morbidity and mortality (1, 2). Patients with autoimmune disease are at an increased risk of osteoarthritis (3–5). Currently, several groups of effective and safe antihistaminic drugs are very often given to treat autoimmune disease (6–8). Antihistamines are primarily used to treat autoimmune diseases and allergic disorders and are not typically associated with the development of osteoarthritis, which is a degenerative joint disease characterized by the breakdown of cartilage and underlying bone. However, there is no direct evidence or widely recognized association between the use of antihistamines and the new onset of osteoarthritis in patients with autoimmune disease.

Osteoarthritis-related complications are important in the etiology. Moreover, because medications may also affect bone health and comorbidities, the effects of medications on bone metabolism and fracture risk in patients with osteoarthritis should not be neglected. An increased incidence of osteoarthritis has been identified as associated with autoimmune disease (3–5). In addition, treatment for autoimmune disease can have negative or positive adverse effects on bone (9–11). Antihistamines have been linked to new-onset osteoarthritis (NOO) in patients with allergic disorders (12–15). However, the impact of these drugs on NOO is unclear in patients with autoimmune disease. Therefore, we designed this study to investigate the risk of NOO in patients receiving antihistamines compared with nonantihistamines in a population-based cohort study using a Taiwan national health insurance database.

## Materials and methods

### Study design and data source

This is a retrospective cohort study. The patient data were obtained from the National Health Insurance (NHI) program, which is a compulsory universal health insurance program in Taiwan and covers approximately 99% of Taiwanese residents (16). The NHI database stores information, including claim forms, and contains patient sex, age, three diagnostic codes, medical expenditures, and prescriptions, such as drug quantity and expenditure, drug dose, operations, and treatments. All personal information was encrypted and deidentified to preserve patient privacy (16).

### Patient population

This study extracted data from the NHI program in Taiwan from January 2012 to December 2022 by using newly diagnosed autoimmune disease codes based on the International Classification of Diseases (ICD), ninth revision, Clinical Modification (CM) (ICD-9-CM) and ICD, tenth revision, CM (ICD-10-CM). Newly diagnosed autoimmune disease was defined as the first time that an autoimmune disease code was available in outpatient or inpatient claim records between January 2012 and December 2022.

This study included adults (aged ≥ 40 years) with an autoimmune disease ICD-9-CM code 279.x, or ICD-10-CM code M32.9, M35.9, M06.9, M05.9, L93.0, G35, M32.10, L81.9, M34.9, and M05.79 who were treated with at least one continuous antihistamine prescription for more than 180 days between January 2012 and December 2022.

**Figure 1:**
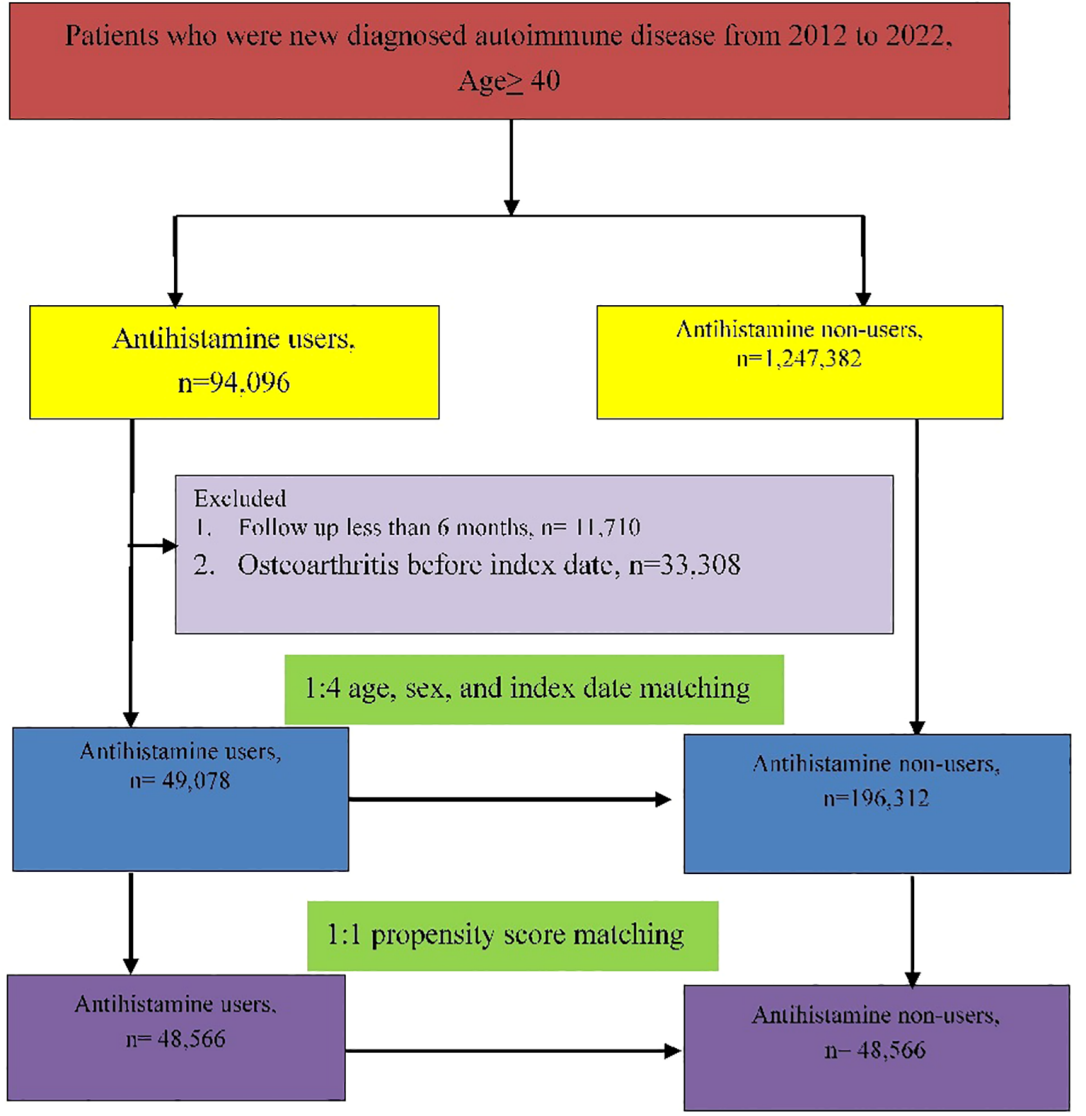
Flowchart of study population.

The participants had to meet at least one of the following criteria: (1) had two or more outpatient visits within 6 months, (2) continuously received antihistamine medication for more than 6 months during the study period, or (3) had one or more inpatient admissions with a diagnosis of autoimmune disease. Comorbidities related to autoimmune disease were recorded in accordance with the ICD-9-CM code, ICD-10-CM code and included coronary artery disease (ICD-9-CM code 414; ICD-10-CM code I20–I25), hypertension (ICD-9-CM code 401-405; ICD-10-CM code I10), diabetes mellitus (ICD-9-CM code 250; ICD-10-CM code E08-E13), congestive heart failure (ICD-9-CM code 428; ICD-10-CM code I50), dementia (ICD-9-CM code 290; ICD-10-CM code F03), hyperlipidemia (ICD-9-CM code 272.x; ICD-10-CM code E78.1–E78.5), chronic liver disease (ICD-9-CM code 571.x; ICD-10-CM codes K71, K75, and K76), stroke (ICD-9-CM code 434; ICD-10-CM codes I60-I69), chronic obstructive pulmonary disease (ICD-9-CM code 490-492, 494-496; ICD-10-CM code J44), asthma (ICD-9-CM code 493; ICD-10-CM code J45), and allergic rhinitis (ICD-9-CM code 477.x; ICD-10-CM code J30.x). Exclusion criteria included(1) a prior history of osteoarthritis before January 2014, (2) follow-up period of less than 6 months, and (3) age less than 40 years. The date of patients who received at least one continuous antihistamine prescription for 180 days was defined as the index date for the antihistamine group. For the non-antihistamine group, the index date corresponded to that of their matched counterparts in the antihistamine group. The antihistamine and non-antihistamine groups were matched for age, sex, and index date at a ratio of 1:4. The final study sample comprised 49,078 antihistamine users and 196,312 non-antihistamine users (**Figure 1**). Sensitivity analysis using propensity score matching was also performed with a matching ratio of 1:1 (**Figure 1**).

### Study variables

Baseline covariates were identified by examining claims records from the NHI program that contained the specified diagnoses or medication codes between 2012 and 2022. Patient demographic characteristics were assessed on the index date. Demographic variables included gender, age, comorbidities, and concurrent medication. Comorbidities closest to the index date within 30 days and medication use were assessed during a 180-day baseline period.

The study endpoint was new-onset osteoarthritis, defined as the first occurrence of an osteoarthritis code (ICD-9-CM code 715, ICD-10-CM codes M15-M19) in inpatient or outpatient claim records during follow-up.

### Statistical methods

Baseline demographic/clinical characteristics were compared between the two study groups. Student’s t-test and chi-square test were used to evaluate the distribution of continuous and categorical variables for a matched cohort. Standardized mean difference ≤ 0.10 indicates a negligible difference in potential confounders between the two cohorts. In both cohorts, the incidence rates of NOO were calculated as per 10,000 person-months. The crude hazard ratios (HRs) and 95% confidence intervals (CIs) of NOO were estimated by using Cox proportional hazard regression. Multivariable models were further adjusted for important risk factors for developing NOO, including comorbidities and concurrent medication. The risk of NOO over time for the antihistamine group compared with that for the non- antihistamine group was determined through survival analysis with the Kaplan–Meier method. Because of the observed differences in baseline characteristics existed, the propensity score matching was performed with a matching ratio of 1:1 to balance baseline covariates between two groups for sensitivity analysis. Subgroup analyses stratified by gender, age, comorbidities, and concurrent medication were performed on the outcomes. Statistical significance was defined at *P*-value < 0.05. All statistical analyses were performed by using SAS 9.4 (SAS Institute Inc., Cary, North Carolina, USA).

## Results

### Patient characteristics

After 1:4 age, sex, and index date matching, we found 49,078 patients on an antihistamine and 196,312 patients not on an antihistamine. The baseline characteristics of all patients in the antihistamine and non-antihistamine groups are presented in **Table 1**. In each group, the majority of patients had males (60.94% versus 39.06%). In both groups, most subjects were 50–59 years of age (29.27%). Compared with non-antihistamine users, antihistamine users had a higher proportion of comorbidities with hypertension, diabetes mellitus, hyperlipidemia, coronary artery disease, congestive heart failure, dementia, rheumatoid arthritis, chronic kidney disease, chronic liver disease, stroke, chronic obstructive pulmonary disease, asthma and allergic rhinitis. The proportion of patients receiving a non-steroidal anti-inflammatory drugs, corticosteroids, and aspirins were higher in those under antihistamines therapy than in those under non-antihistamines therapy.

**Table 1:** Baseline characteristics.

	4:1 sex, age, and index matching			After PSM		
	Antihistamine non-users	Antihistamine users	ASD	Antihistamine non-users	Antihistamine users	AS0D
N	196312	49078		48566	48566	
Sex			0.0000			0.0075
Female	76680 (39.06%)	19170 (39.06%)		18763 (38.63%)	18940 (39.00%)	
Male	119632 (60.94%)	29908 (60.94%)		29803 (61.37%)	29626 (61.00%)	
Age			0.0000			0.0000
40-49	53788 (27.40%)	13440 (27.38%)		12963 (26.69%)	13275 (27.33%)	
50-59	57467 (29.27%)	14336 (29.21%)		14040 (28.91%)	14181 (29.20%)	
60-69	42727 (21.76%)	10692 (21.79%)		10782 (22.20%)	10585 (21.80%)	
>=70	42330 (21.56%)	10610 (21.62%)		10781 (22.20%)	10525 (21.67%)	
Comorbidity						
Hypertension	61503 (31.33%)	17937 (36.55%)	0.1104	18221 (37.52%)	17679 (36.40%)	0.0231
Diabetes mellitus	31821 (16.21%)	10872 (22.15%)	0.1514	10809 (22.26%)	10626 (21.88%)	0.0091
Hyperlipidemia	41215 (20.99%)	11454 (23.34%)	0.0565	11724 (24.14%)	11291 (23.25%)	0.0210
CAD	16407 (8.36%)	4627 (9.43%)	0.0376	4660 (9.60%)	4575 (9.42%)	0.0060
Congestive heart failure	4796 (2.44%)	1824 (3.72%)	0.0738	1688 (3.48%)	1784 (3.67%)	0.0107
Dementia	4541 (2.31%)	1621 (3.30%)	0.0599	1503 (3.09%)	1590 (3.27%)	0.0102
Chronic kidney disease	12396 (6.31%)	4991 (10.17%)	0.1405	4660 (9.60%)	4821 (9.93%)	0.0112
Chronic liver disease	16373 (8.34%)	5442 (11.09%)	0.0929	5550 (11.43%)	5303 (10.92%)	0.0161
Stroke	8362 (4.26%)	2884 (5.88%)	0.0738	2771 (5.71%)	2827 (5.82%)	0.0050
COPD	11393 (5.80%)	4291 (8.74%)	0.1134	4389 (9.04%)	4231 (8.71%)	0.0114
Asthma	6964 (3.55%)	2931 (5.97%)	0.1141	2990 (6.16%)	2885 (5.94%)	0.0091
Allergic rhinitis	18284 (9.31%)	9539 (19.44%)	0.2916	9572 (19.71%)	9109 (18.76%)	0.0242
Medication						
NSAIDs	117283 (59.74%)	30897 (62.95%)	0.0660	31775 (65.43%)	30616 (63.04%)	0.0498
Corticosteroids	45526 (23.19%)	30648 (62.45%)	0.8643	30228 (62.24%)	30136 (62.05%)	0.0039
Aspirin	24349 (12.40%)	6986 (14.23%)	0.0539	6990 (14.39%)	6914 (14.24%)	0.0045

ASD, absolute standardized difference; CAD, coronary artery disease; COPD, chronic obstructive pulmonary disease; NSAIDs, non-steroidal anti-inflammatory drugs; PSM, propensity score matching.

### NOO during follow-up

**Figure 2:**
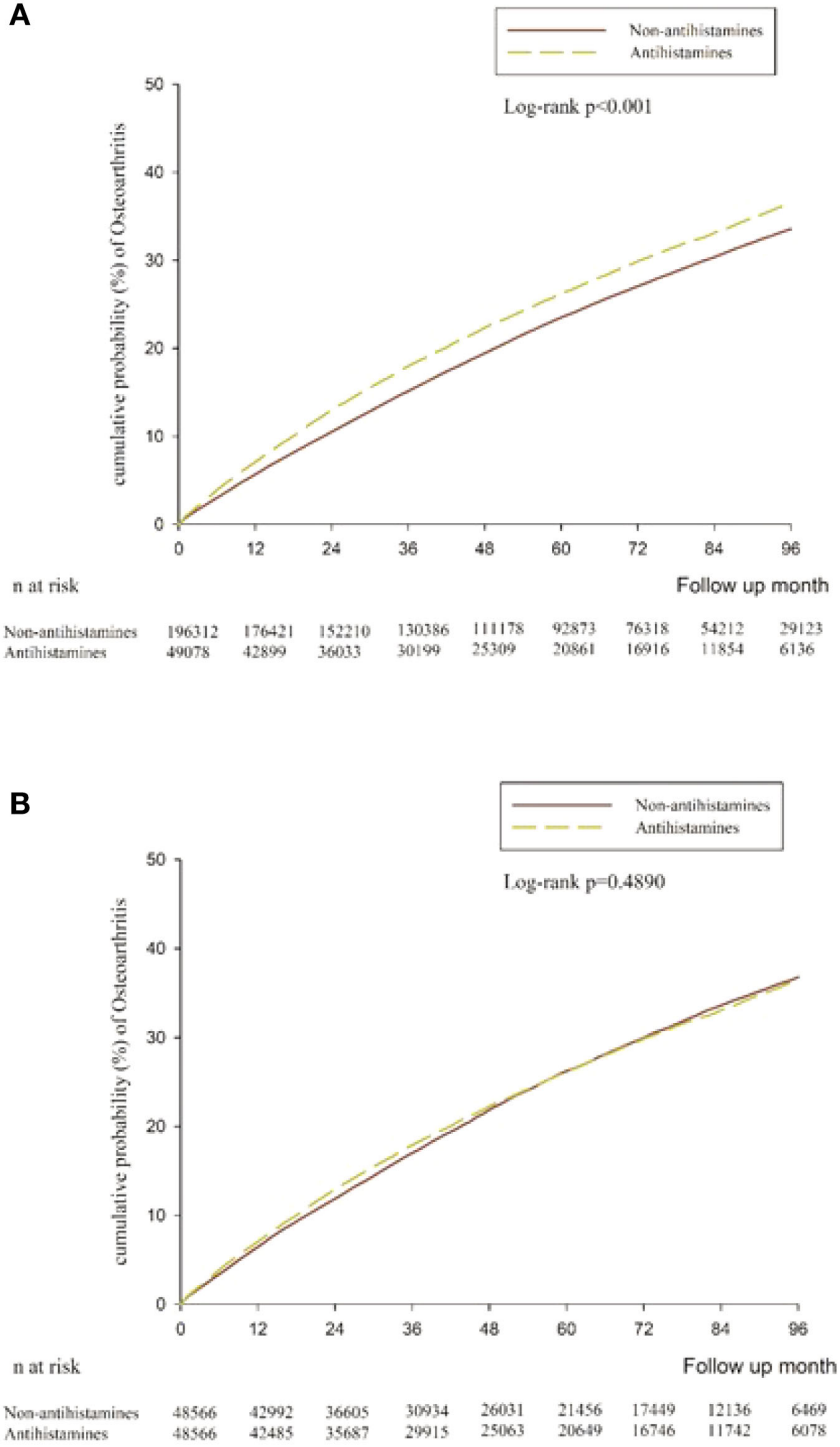
**(A)** Cumulative risk curve of new-onset osteoarthritis for the study cohorts treated with antihistamine versus non-antihistamine users. **(B)** Cumulative risk curve of new-onset osteoarthritis for the study cohorts under propensity score matching treated with antihistamine versus non-antihistamine users.

The follow-up periods were 2,555,371 person-months in the antihistamine group and 10,972,161 person-months in the non-antihistamine group. The patients with antihistamine treatment had a higher incidence rates of NOO than the patients not receiving antihistamine treatment (4.99 versus 4.37 per 1,000 person-months). The antihistamine group showed a significant association with a higher risk of NOO (HR 1.14, 95% CI 1.12–1.16, *P* < 0.0001) (**Table 2**). After adjusting for variables including sex, age, comorbidities and concurrent medication at baseline, the risk of NOO still increased in the antihistamine user compared to the antihistamine non-user (HR 1.05, 95% CI 1.03–1.07, P < 0.0001) (**Table 2**). The result after Kaplan–Meier analysis also demonstrated that the cumulative probability of NOO was significantly higher in patients with antihistamine prescription (*P* < 0.001) (**Figure 2A**).

**Table 2:** Incidence rate in study groups.

	4:1 sex, age, and index date matching			After PSM		
	Antihistamine non-users	Antihistamine users	*P* value	Antihistamine non-users	Antihistamine users	*P* value
N	196312	49078		48566	48566	
Follow up person months	10972161	2555371		2599400	2530380	
New case	47982	12761		12839	12607	
Incidence rate*(95% CI)	4.37(4.33-4.41)	4.99(4.91-5.08)		4.94(4.85-5.03)	4.98(4.90-5.07)	
Crude Relative risk (95% CI)	reference	1.14(1.12-1.16)	<.0001	reference	1.01(0.98-1.03)	0.5767
Adjusted HR (95% CI)†	reference	1.05(1.03-1.07)	<.0001	reference	1.04(1.01-1.06)	0.0058

Incidence rate: per 1,000 person-months.

CI, confidence interval; HR, hazard ratio; PSM, propensity score matching.

†adjusted hazard ratio, the covariates including year of index, sex, age, co-morbidities and medication at baseline.

### Sensitivity and subgroup analysis

We performed sensitivity analysis on participants with a propensity score 1:1 matching analysis. The association between antihistamine use and NOO incidence did not change when compared with the main results (aHR: 1.04, 95% CI 1.01–1.06, **Table 2**; or **Figure 2B**).

The subgroup analysis were partly consistent with the main analyses between the antihistamine group and the non-antihistamine group (**Table 3**). The results were also consistent with the findings of the main analyses in the age ≥ 50 versus <50, comorbidity with hypertension, hyperlipidemia, coronary artery disease, chronic liver disease, chronic obstructive pulmonary disease, asthma, and allergic rhinitis, and concurrent medication with non-steroidal anti-inflammatory drug, corticosteroid and aspirin. However, among antihistamine users, males were at significantly lower risk for NOO than females (aHR: 0.75; 95% CI 0.74–0.76). Moreover, comorbidity with dementia and stroke in the antihistamine user group had a significant lower risk of NOO compared to those in the antihistamine nonuser group (dementia, aHR: 0.75, 95% CI 0.71–0.80; stroke, aHR: 0.92, 95% CI 0.88–0.95 in **Table 3**).

**Table 3:** Multiple Cox regression to estimate the hazard ratio.

	4:1 sex, age, and index date matching	1:1 PSM
	aHR (95% C.I.)†	aHR (95% C.I.)†
Antihistamines		
Antihistamine non-users	reference	reference
Antihistamine users	1.05(1.03-1.07)	1.04(1.01-1.06)
Sex		
Female	reference	reference
Male	0.75(0.74-0.76)	0.74(0.72-0.76)
Age		
40-49	reference	reference
50-59	1.55(1.51-1.59)	1.53(1.48-1.59)
60-69	2.22(2.16-2.28)	2.17(2.09-2.26)
>=70	2.75(2.68-2.83)	2.45(2.35-2.56)
Comorbidity		
Hypertension	1.11(1.09-1.13)	1.11(1.08-1.14)
Diabetes mellitus	0.96(0.94-0.98)	0.97(0.94-1.00)
Hyperlipidemia	1.15(1.13-1.17)	1.15(1.11-1.18)
CAD	1.16(1.13-1.20)	1.16(1.11-1.21)
Congestive heart failure	0.99(0.94-1.03)	1.02(0.95-1.09)
Dementia	0.75(0.71-0.80)	0.70(0.65-0.77)
Chronic kidney disease	1.03(1.00-1.06)	1.04(0.99-1.08)
Chronic liver disease	1.17(1.14-1.20)	1.15(1.11-1.19)
Stroke	0.92(0.88-0.95)	0.92(0.87-0.97)
COPD	1.08(1.05-1.12)	1.08(1.03-1.12)
Asthma	1.05(1.01-1.09)	1.07(1.02-1.13)
Allergic rhinitis	1.17(1.14-1.20)	1.15(1.11-1.18)
Concurrent medication		
NSAIDs	1.47(1.44-1.49)	1.47(1.43-1.51)
Corticosteroids	1.16(1.13-1.18)	1.12(1.09-1.15)
Aspirin	1.02(0.99-1.04)	1.04(1.00-1.08)

CAD, coronary artery disease; CI, confidence interval; COPD, chronic obstructive pulmonary disease; HR, hazard ratio; NSAIDs, non-steroidal anti-inflammatory drugs; PSM, propensity score matching.

## Discussion

In this population-based cohort study, we observed that patients taking antihistamines had a significantly higher risk for NOO than nonantihistamine users did in patients with autoimmune disease. The Kaplan–Meier and sensitivity analyses also demonstrated a significantly higher risk for NOO among patients in the antihistamine group. However, the results of the subgroup analyses in this study indicated a decreased risk of osteoarthritis incidence in male and young (< 50 years) patients.

The association between antihistamine use and incident osteoarthritis supports the findings of mechanistic studies that implicated a number of allergic pathways in the pathogenesis of osteoarthritis: (1) activated and degranulating mast cells are statistically increased in the synovium of patients with osteoarthritis (17), and there is a nonsignificant trend between the numbers of synovial mast cells and the severity of osteoarthritis (18); (2) mast cell activation and the release of tryptase mediate synovitis and the breakdown of joint tissue in osteoarthritis (17, 19, 20); and (3) genomic analyses identified associations between interleukin-4 (IL-4) and IL-4 receptor gene polymorphisms and the development of osteoarthritis (21–24). Furthermore, increased mast cell numbers and elevated tryptase levels have also been involved in the pathogenesis of asthma (25). Taken together, we hypothesize that pathways may contribute to antihistamine use associated with a significantly increased risk of osteoarthritis.

Consistent with the results reported in the literature, our findings showed a significant increase in the risk of developing NOO among patients taking antihistamines (12). Turkiewicz et al. evaluated 25,003 patients and found that the risks for the subsequent development of NOO were increased among antihistamine users as compared with antihistamine nonusers. However, an analysis of a clinical trial from the osteoarthritis initiative showed that antihistamine use might have a more favorable effect on NOO outcomes than the nonuse of antihistamines (13, 14). Another study also reported that antihistamine use was more effective for preventing NOO than antihistamine nonuse was (15). The difference of results between our study and previous studies is due to differences in study population, medication type/dosage, and follow-up duration (13–15). Therefore, it remains uncertain whether the risk of NOO differs among antihistamine users as compared with antihistamine nonusers. However, the impact of these antihistamines on NOO is unclear in patients with autoimmune disease till now. To our knowledge, this is the first study conducted within a Taiwan NHI program setting exploring the association between antihistamines and NOO following an autoimmune disease diagnosis.

Our study found a gender difference between the antihistamine and nonantihistamine users in terms of the incidence of NOO in patients with autoimmune disease. Previous studies have demonstrated gender differences in the incidence of osteoarthritis across a variety of study populations (26–29). Di et al. analyzed differences in region, age, and sex with regard to the incidence of knee osteoarthritis from the GBD study for 204 nations and territories between 1990 and 2019. After adjusting for baseline differences, they found that women were more likely than men to experience knee osteoarthritis (27). Many known risk factors for osteoarthritis are female gender: (1) women older than 50 years are more likely to develop osteoarthritis than men are; (2) women often report more severe symptoms and a higher degree of functional impairment compared with men; (3) gender related hormone levels, particularly those related to menopause, are thought to contribute to the increased risk of osteoarthritis in women; and (4) females have been shown to seek more health care input from hospitals, which could lead to more recorded diagnoses (30–32). These gender differences highlight the importance of considering sex-specific factors in NOO.

Most clinical trials have reported that old age is clearly a risk factor for developing osteoarthritis (33, 34). In the present study, antihistamine use resulted in a significantly deleterious effect against the NOO in users aged ≥ 50 years relative to in users aged < 50 years. The age-related differences in antihistamine use can be attributed to differences in longevity, survival bias, and comorbidities between older and young patients (35). Another reason is that older individuals are more likely to report having joint injury, obesity, genetic, and anatomical factors that affect joint mechanics (36). To elucidate the mechanisms underlying this association, further comprehensive clinical research is warranted.

Interestingly, this study showed that antihistamine users were significantly associated with decreased NOO risk for patients with dementia/stroke comorbidities in subgroup analysis. Antihistamine drugs used to treat autoimmune disease bind to the H-1 receptor. Of the known histamine receptors, the H1-receptor is believed to be most clearly associated with potentiation of proinflammatory immune cell activity (37). Histamine demonstrated regulating the sleep-waking cycle and promoting cognition and memory (38). Targeted disruption of the histamine H1 receptor gene *in vivo* study contributes to the impairment of memory function (39). This might be partially explained by our results. The pathophysiological mechanisms underlying these difference in dementia/stroke comorbidities remain unclear. Thus, long-term prospective studies are needed to clarify our findings.

The strengths of our study include its population-based nature, large sample size, and the use of real-world data. However, our study also has several limitations. First, the retrospective data–based analysis carries certain inherent limitations. Second, some laboratory data, such as fasting blood sugar levels, renal function, and liver function, as well as some imaging findings were not available from the NHI data. However, because the data were population based, we assumed that there were no differences between the two groups. Third, other residual confounding factors, such as genetic, physical activity, or dietary factors, were also not included in the NHI data. Further randomized clinical trials are needed to confirm our results. Fourth, the study population is only autoimmune disease patients aged 40 and above in Taiwan, and the results are difficult to directly extrapolate to other regions or young populations due to regional factors such as genetic background, medical system, and medication habits. Therefore, our findings may not be generalizable to patients in other countries.

In conclusion, the prescription of an antihistamine increased the risk of NOO in patients with autoimmune disease, and this risk was greater among females and those older than 50 years. To explore the difference between antihistamine prescription and the incidence of osteoarthritis, further comprehensive clinical research may be needed, which could allow us to develop innovative strategies and novel therapies with the purpose of preventing new disease onset in patients with autoimmune disease.

## Data Availability

The original contributions presented in the study are included in the article/supplementary material. Further inquiries can be directed to the corresponding authors.
